# Clinical and Microbiological Characteristics of Group B *Streptococcus* from Pregnant Women and Diseased Infants in Intrapartum Antibiotic Prophylaxis Era in Taiwan

**DOI:** 10.1038/s41598-019-49977-2

**Published:** 2019-09-19

**Authors:** Chien-Chung Lee, Jen-Fu Hsu, Rajendra Prasad Janapatla, Chyi-Liang Chen, Ying-Li Zhou, Reyin Lien, Cheng-Hsun Chiu

**Affiliations:** 1 Division of Neonatology, Department of Pediatrics, Chang Gung Memorial Hospital, Chang Gung University College of Medicine, Taoyuan, Taiwan; 2 Molecular Infectious Disease Research Center, Chang Gung Memorial Hospital, Taoyuan, Taiwan; 3 Division of Pediatric Infectious Diseases, Department of Pediatrics, Chang Gung Memorial Hospital, Chang Gung University College of Medicine, Taoyuan, Taiwan

**Keywords:** Clinical microbiology, Microbial genetics

## Abstract

Group B *Streptococcus* (GBS) is one of the most important pathogens for neonates. This study included 69 invasive GBS diseases in neonates, including 7 early-onset disease (EOD), 55 late-onset disease, and 7 very-late-onset disease from 2013 to 2017. A significant reduction of EOD after the deployment of intrapartum antibiotic prophylaxis (IAP) in 2012 was observed. A previously-recognized hypervirulent clone GBS III ST17, accounting for 68% of the overall infections and 71% of the meningitis, was identified among the 69 cases. A novel GBS Ia ST890 emerged, becoming the fourth most common clone. Overall 96% of the invasive GBS infections were caused by serotypes Ia, Ib, and III. We collected 300 GBS isolates from vagina of the healthy pregnant women in 2014 and 2017. The serotype distribution of the maternal colonization isolates was VI (35%), III (21%), V (15%), Ib (13%) and Ia (11%) in 2014, and VI (32%), III (22%), V (16%), Ia (16%), and Ib (8%) in 2017. The most common sequence types were ST1 (32%), ST12 (22%), and ST23 (15%). Serotype diversity of maternal colonization strains did not change between 2014 and 2017. The study provides useful information in surveillance of GBS disease in the era of IAP.

## Introduction

Group B *Streptococcus* (GBS) often colonizes the gastrointestinal and genital tract of 15–40% of pregnant women, and can cause GBS infection in neonates and young infants^1,2^. GBS infection in infants includes sepsis, pneumonia, meningitis, bacteraemia, or focal infections, and can translate to significant mortality^3,4^. GBS infections, according to timing, are further divided into early-onset (EOD; birth to 6 days), late-onset (LOD; 7 to 89 days), and very-late-onset disease (VLOD; older than 3 months of age). Although the implementation of intrapartum antibiotic prophylaxis (IAP), the incidence of EOD declined from 1.8 to 0.24 cases per 1000 live births, whereas the incidence of LOD has remained between 0.3 and 0.4 per 1000 live births in the United States^5^. GBS infections remain a burden of neonatal sepsis worldwide that estimation of EOD incidence was 0.41 per 1000 live births, LOD incidence was 0.26, case fatality risk (CFR) was 8.4%^6^. In Taiwan, according to a previous cohort study involving 5 medical centres and 6 regional hospitals, maternal GBS colonisation was around 20%, and the mean incidence of GBS disease was 1.1 per 1000 live births. The CFR was 8.4% for EOD diseases and 3.8% for LOD^7^.

GBS based on capsular polysaccharide can be classified into 10 serotypes (Ia, Ib, and II through IX). The serotype distribution of GBS varies in different geographic regions. The overall GBS infections were most commonly caused by Ia, Ib, II, III, and V which accounted for more than 85% of the serotypes. In Taiwan, IAP was introduced in 2010 and GBS screening for all pregnant women was introduced in 2012. However, much remains unknown about the distribution of the serotypes and genotypes of maternal GBS colonisation isolates, and those of invasive GBS diseases isolates in infants in the era of IAP. This study was carried out to fill this information gap.

## Results

### Clinical manifestations of invasive GBS disease in infants

From January 2013 to June 2017, there were 68 infants with 69 episodes of invasive GBS diseases, and their clinical manifestations are shown in Table 1. Among these GBS infections, 7 (10%) were EOD, 55 (80%) were LOD, and 7 (10%) were VLOD. One infant got GBS sepsis at the age of 18 days and 32 days, respectively. Among 7 infants with EOD, three were premature infants with unknown maternal GBS status; another four full-term infants were born with negative maternal GBS screens. Interestingly, one mother had initially negative GBS carriage at 35-week pregnancy, but become positive after rechecking maternal GBS carriage because we found that her 38-week baby developed EOD at two days old. Among 7 infants that had VLOD, 4 infants were younger than 6 months of age. Comparing the EOD to LOD invasive GBS disease, although not statistically significant, there were more premature infants in the EOD group (42.9% *vs*. 14.5%, *P* = 0.099). Infants with EOD were more prone to develop respiratory failure (71.4% *vs*. 3.2%, *P* < 0.001), whereas infants with LOD invasive GBS disease were more prone to have fever (94.5% *vs*. 28.6%, *P* < 0.001). Only 42.9% of infants with EOD had leukocyte abnormality, and 28.6% had CRP elevations. Similarly, only 25.5% and 23.6% of infants with LOD had leukocyte abnormality and CRP elevation, respectively. Neither leukocyte abnormality nor elevation of CRP level on the initial hemogram test can be reliably used to identify invasive GBS diseases. Twelve infants developed meningitis, though only seven episodes of meningitis were culture-proven. Other 5 meningitis cases were judged based on positive GBS antigen and pleocytosis in cerebrospinal fluid analysis. Clinical characterizations of infants with and without meningitis were compared in Table 2. Infants who developed meningitis were more likely to manifest altered level of consciousness (66.7% *vs*. 33.9%, *P* = 0.018), seizure (50% *vs*. 24.6%, *P* < 0.001), and CRP elevation (91.7% *vs*. 45.6%, *P* = 0.004). Only one infant who developed early-onset GBS sepsis and meningitis (not culture-proved) died.

**Table 1:** Clinical characteristics of invasive GBS infections.

Groups	EOD n = 7	LOD n = 55	VLOD n = 7	*P1*	*P2*	*P3*
Male, (%)	5 (71.4)	20 (36.4)	6 (85.7)	0.107	1	0.018
Prematurity, (%)	3 (42.9)	8 (14.5)	1 (14.3)	0.099	0.556	1
Vaginal delivery, (%)	6 (85.7)	38 (69.1)	3 (42.9)	0.437	0.266	0.214
Onset age (median, IQR)	1 (1,3)	44 (22, 54)	140 (115, 205)	<0.001	<0.001	<0.001
Survival, (%)	6 (85.7)	55(100)	7 (100)	0.113	1	1
Meningitis, (%)	2 (28.6)	9 (16.4)	1 (14.3)	0.597	1	1
Leukocyte counts (10^3^/μL)^¶^, (mean ± SD)	10.9 ± 10.7	10.9 ± 6.8	14.1 ± 7.5	0.939	0.537	0.211
Leukocyte abnormality^¶,†^ (%)	3 (42.9)	14 (25.5)	3 (42.9)	0.381	1	0.381
CRP level (mg/mL)^¶^ (mean ± SD)	67.2 ± 120	17.3 ± 24.5	43 ± 69.6	0.314	0.652	0.369
CRP elevation^¶,‡^ (%)	2 (28.6)	13 (23.6)	2 (28.6)	1	1	1
Fever (%)	2 (28.6)	52 (94.5)	5 (71.4)	<0.001	0.286	0.093
Lethargy or irritability, (%)	3 (42.9)	18 (32.7)	3 (42.9)	0.68	1	0.68
Respiratory failure^§^, (%)	5 (71.4)	2 (3.6)	0	<0.001	0.021	1
Seizure, (%)	2 (28.6)	6 (10.9)	1 (14.3)	0.22	1	1

EOD: early-onset disease, LOD: late-onset disease, VLOD: very-late-onset disease,

IQR: interquartile range, SD: standard deviation.

^¶^The first hemogram study during hospitalization.

^†^White blood cells <4,000/*μL* or > 20,000/*μL*, ^‡^CRP > 20 *mg*/*dL*.

^§^Need for intubation and ventilator support.

*P1* is evaluated by the comparison between EOD and LOD,

*P2* is evaluated by the comparison between EOD and VLOD.

*P3* is evaluated by the comparison between LOD and LOD.

**Table 2:** Clinical characteristics of invasive GBS infections with and without meningitis.

Groups	Meningitis n = 12	No meningitis n = 57	*P*
Male, (%)	4 (33.3)	27 (47.4)	0.374
Prematurity, (%)	1 (8.3)	11 (19.3)	0.677
Vaginal delivery, (%)	9 (75)	38 (66.7)	0.622
Onset age (median, IQR)	40 (10, 62)	44 (22, 55)	0.879
Survival, (%)	11 (91.7)	57 (100)	0.174
Leukocyte counts (10^3^/μL)^¶^, (mean ± SD)	9.7 ± 8	11.2 ± 7.1	0.519
Leukocyte abnormality^¶,†^ (%)	6 (50)	14 (24.6)	0.092
CRP level (mg/mL)^¶^ (mean ± SD)	89 ± 88	11.5 ± 19.5	0.011
CRP elevation^¶,‡^ (%)	9 (75)	8 (14)	<0.001
Fever (%)	12 (100)	47 (82.5)	0.191
Altered level of consciousness (%)	8 (66.7)	21 (33.9)	0.018
Respiratory failure^§^, (%)	1 (8.3)	6 (10.5)	1
Seizure, (%)	6 (50)	14 (24.6)	<0.001

EOD: early-onset disease, LOD: late-onset disease, VLOD: very-late-onset disease,

IQR: interquartile range, SD: standard deviation.

^¶^The first hemogram study during hospitalization.

^†^White blood cells <4,000/*μL* or >20,000/*μL*, ^‡^CRP > 20 *mg*/*dL*.

^§^Need for intubation and ventilator support.

### Serotypes of clinical isolates

**Figure 1:**
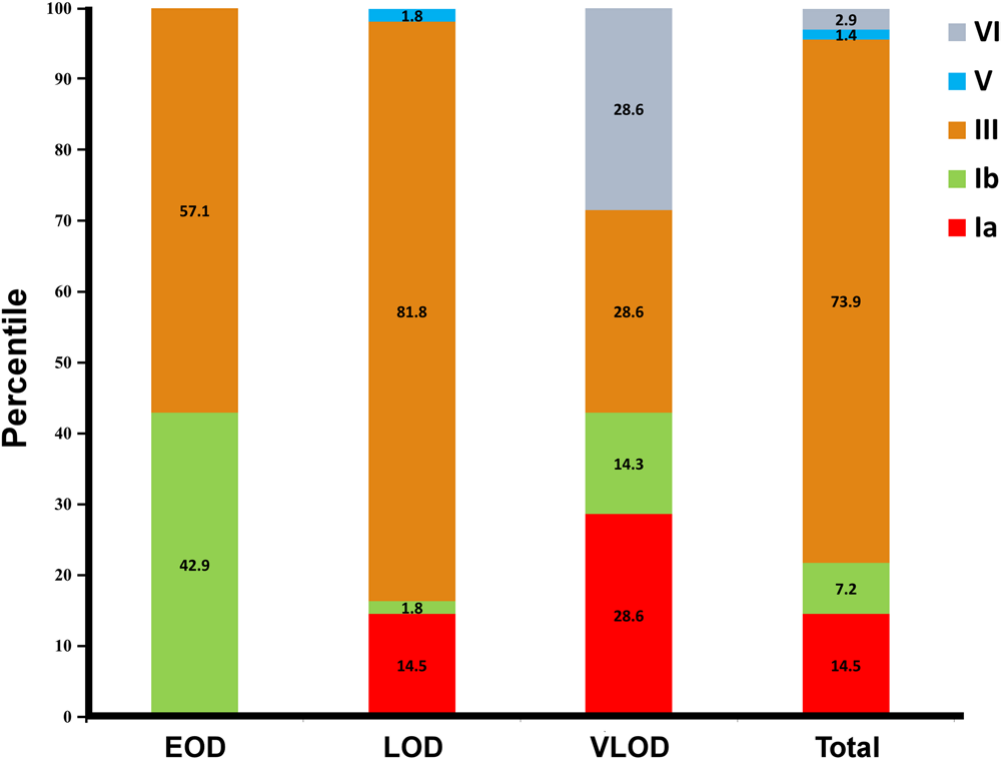
Serotype distribution of invasive GBS isolates in infants. The colors representing different serotypes are shown on the right-side color scheme, and the numbers in the color bar represent the percentage of serotypes for the disease shown at the bottom. EOD: early-onset disease, LOD: late-onset disease, VLOD: very-late-onset disease.

The serotype distributions of invasive GBS infections are shown in Fig. 1. Among all GBS infections, GBS III (74%) was the most predominant clone, followed by Ia (15%), Ib (7%), VI (3%), and V (1%). According to the onset timing of the diseases, GBS III (57%) and Ib (43%) caused EOD, whereas GBS III (82%), Ia (15%), Ib (2%), and V (2%) accounted for LOD, and GBS III (29%), Ia (29%), VI (29%), and Ib (14%) contributed to VLOD. The molecular characteristics of invasive GBS diseases are summarized in Table 3 and Supplementary Fig. S1. The most common sequence types were ST17 (68%), ST23 (9%), and ST12 (7%). A hyper-virulent clone, GBS III ST17, accounted for 68% of the invasive GBS infections. Next, GBS Ia ST23 and GBS Ib ST12, each accounted for 7% of GBS infections. A novel clone GBS Ia ST890 emerged as the fourth common pathogen and caused 4% of the invasive GBS diseases. Notably, the two genotypes of LOD invasive isolates were not identified in maternal GBS colonisation. One was GBS III ST578, and the other was allelic profile identified from GBS III (new 3) which was not registered before. In clinical manifestations, five of seven (71%) culture-proved meningitis cases were caused by GBS III ST17, one was by GBS III ST19, and the other was by GBS Ia ST23. Furthermore, the infant who had GBS infections at 18 days and 32 days of age showed GBS III ST17, and the infant who died was infected with GBS Ib ST12.

**Table 3:** Microbiological characteristics of invasive GBS isolates in infants and colonizing GBS isolates in pregnant women.

ST	Allele numbers of housekeeping genes	Invasive GBS isolates		Colonizing GBS isolates	
		n = 69 (%)	Serotype (n)	n = 60 (%)	Serotype (n)
ST1	1, 1, 2, 1, 1, 2, 2	3 (4%)	V (1) VI (2)	19 (32%)	V (9) VI (10)
ST10	9, 1, 4, 1, 3, 3, 2	0	—	1 (2%)	Ib (1)
ST12	10, 1, 4, 1, 3, 3, 2	5 (7%)	Ib (5)	13 (22%)	Ib (13)
ST17	2, 1, 1, 2, 1, 1, 1	47 (68%)	III (47)	4 (7%)	III (4)
ST19	1, 1, 3, 2, 2, 2, 2	1 (1%)	III (1)	3 (5%)	III (2) V (1)
ST23	5, 4, 6, 3, 2, 1, 3	6 (9%)	Ia (5) III (1)	9 (15%)	Ia (8) III (1)
ST24	5, 4, 4, 3, 2, 3, 3	2 (3%)	Ia (2)	3 (5%)	Ia (2) V (1)
ST335	1, 1, 43, 2, 2, 2, 2	0	—	2 (3%)	III (2)
ST578	2, 1, 6, 2, 1, 1, 1	1 (1%)	III (1)	0	—
ST651	16, 1, 6, 70, 9, 9, 2	0	—	2 (3%)	III (2)
ST890	5, 4, 4, 3, 9, 3, 3	3 (4%)	Ia (3)	2 (3%)	Ia(1) V (1)
New 1	1, 1, 12, 1, 1, 2, 2	0	—	1 (2%)	VI (1)
New 2	56, 1, 3, 4, 9, 2, 5	0	—	1 (2%)	III (1)
New 3	2, 1, 1, 2, 1, 1, 35	1 (1%)	III (1)	0	—

New 1, 2 and 3 are new sequencing type not registered before.

### Maternal GBS colonization

**Figure 2:**
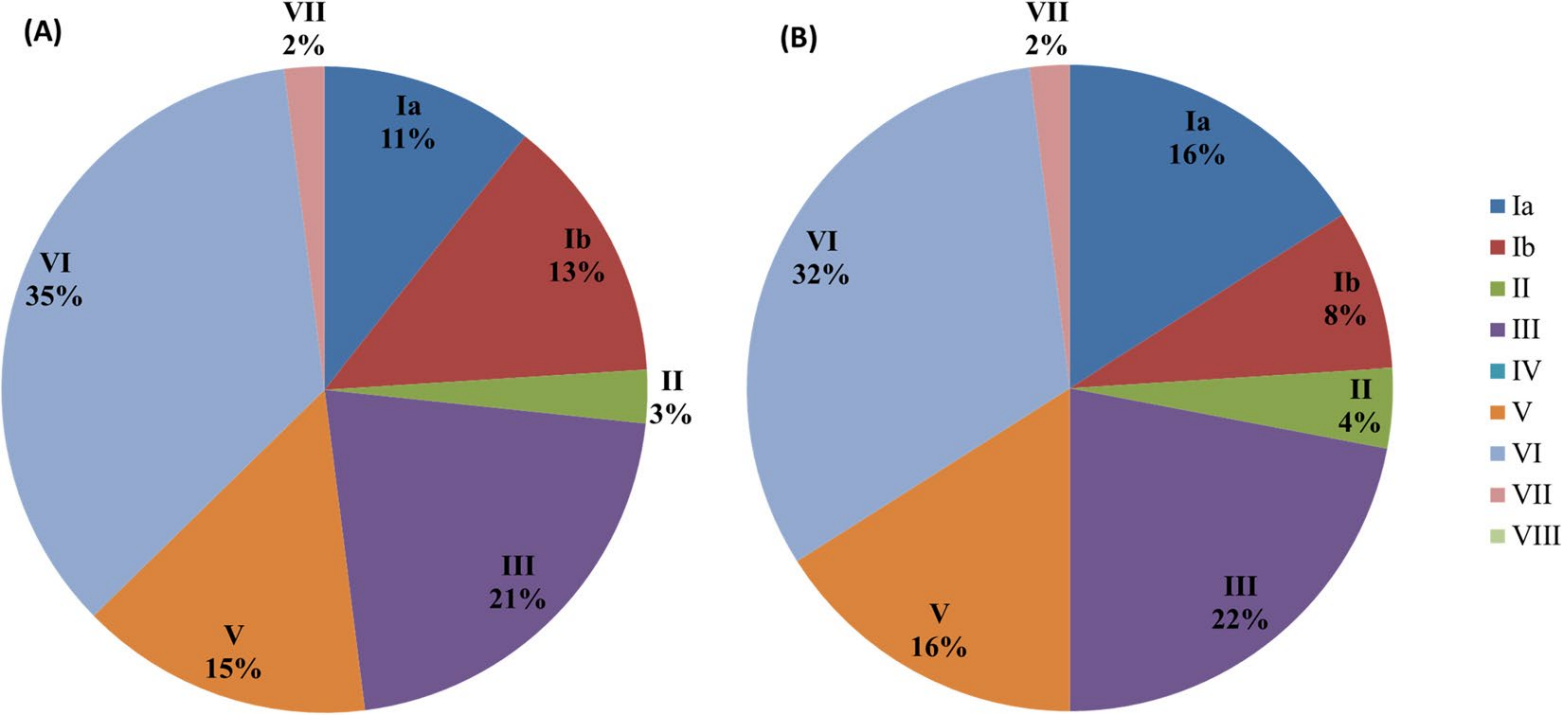
Serotype distribution of GBS strains colonizing in pregnant women. (**A**) GBS isolated from 150 pregnant women in 2014; and (**B**) GBS isolated from 150 pregnant women in 2017.

The molecular characteristics of maternal GBS colonisation is shown in Fig. 2 and Table 3. In 2014, the five most common colonizing GBS clones in pregnant women were VI (53/150, 35%), III (32/150, 21%), V (22/150, 15%), Ib (20/150, 13%), and Ia (16/150, 11%). In 2017, the leading colonizing GBS clone in pregnant women was VI (48/150, 32%), followed by III (33/150, 22%), V (24/150, 16%), Ia (24/150, 16%), and Ib (12/150, 8%). There was no notable variation in serotype distributions between 2014 and 2017. Unlike invasive GBS strains in infants, the colonized strains in pregnant women had more genetic diversity, and the most common sequence types were ST1 (32%), ST12 (22%), and ST23 (15%). Two allelic profiles identified from GBS VI (new 1) and GBS III (new 2) were not registered before. There was no difference in genotype distribution between 2014 and 2017.

## Discussion

In our study, only 7 (10%) GBS disease belonged to EOD in the era of IAP in Taiwan. This is compatible to the result from a recent cohort study in Taiwan, showing that after screening of 9,535 pregnant women and their live born neonates from 2011 to 2016, the incidence rate of EOD declined from 2.8 cases per 1000 live births (%) to nearly zero in 2016, but the LOD incidence rate remained approximately 0.7% before and after the maternal GBS screening^8^. Notably, 17.4% of all GBS diseases, including 42.9% of EOD, 14.5% of LOD and 14.3% of VLOD, were contributed by prematurity birth. Because GBS screening of pregnant women was performed between 35–37 weeks of gestation, prematurity has become a significant risk factor to develop EOD with an OR 4.22 (95% CI 2.04–8.73) in Taiwan^8^. Indeed, in a nationwide surveillance by the Centers for Disease Control of and Prevention of United States from 2005 to 2014, incidence of GBS EOD was an order of magnitude higher among infants <1500 *g* or infants <34 weeks of gestational age compared with infants born at term^9^.

Early diagnosis of neonatal sepsis is extremely challenging due to the lack of accurate biomarkers, nonspecific signs and symptoms, and involvement of all organs and systems^10–13^. We found that infants with EOD are prone to develop respiratory failure requiring intubation and ventilator support. Unlike 94.5% infants with LOD GBS infections that presented with fever, less than 30% of the infants with EOD had fever. These clinical features are similar to those of previous large cohort studies^7,8^. In another large cohort study on 389 infants with EOD from the National Institutes of Health in the U.S., 310 (80%) infants were symptomatic, and 297 (76%) infants had respiratory distress^14^. Moreover, we found that the white blood cell count and CRP level were not reliable biomarkers for invasive GBS infections for both EOD and LOD. These clinical and laboratory characters made it hard to early differentiate neonatal sepsis from other systemic illness. Highly alert for high risk infants and early intervention is still crucial to prevent neonatal sepsis. On the other hand, infants with sepsis who had CRP elevation, as well as altered level of consciousness, and seizures, should raise a suspicion for meningitis.

In Taiwan, few studies reported serotype distributions of invasive GBS disease in infants. A small study enrolled 54 children with GBS disease in southern Taiwan from 1991 to 2002, wherein serotypes were screened for 34 isolates (3 isolates of EOD, 18 isolates of LOD, and 13 isolates of VLOD), and GBS III emerged as the most common serotype accounting for 56% of the isolates^15^. One recent study included 8 colonizing isolates and 126 (17 from young infants) invasive isolates in central Taiwan from 2007 to 2010, GBS VI was the leading cause of EOD, and GBS III was more found in LOD infants^16^. In our recent study, GBS Ia and GBS III were the most predominant clones of EOD before the era of IAP^17^. However, in this study, EOD was caused by GBS III (57%) and GBS Ib (43%). The GBS III, Ia, and Ib accounted for 96% of invasive GBS infections. The contribution of GBS Ia, Ib and III in Taiwan was higher than the worldwide average, but similar to that in Japan. According to one meta-analysis of serotype prevalence including 6500 bacterial isolates, the worldwide serotype distribution was GBS III (61.5%), Ia (19.1%), Ib (5.7%), V (6.7%), II (3.9%) and IV (1.1%)^6^. In a nationwide surveillance study from 2011 to 2015 in Japan, 132 isolates from 445 infants with invasive GBS infections were serotyped, and GBS III (63, 48%), Ia (48, 36%), and Ib (13, 10%) were responsible for 94% of the pathogens determined^18^. This suggests that a trivalent GBS vaccine targeting Ia, Ib, and III will have a good cost-benefit ratio and could prevent 96% of invasive GBS diseases in Taiwan.

In our study, a hyper-virulent clone GBS III ST17, accounted for 68% of invasive GBS diseases and 71% of culture-proven meningitis. However, it only accounted for 7% of maternal GBS colonisation. Previous MLST studies have identified a hyper-virulent GBS clonal complex 17 (CC17)^19^, exclusively comprised of ST17 and ST291. ST17 differs from ST291 by a single nucleotide polymorphism in *pheS*, one of the seven housekeeping genes used for sequence typing; however, the remaining six loci are the same in the two STs. ST17 belonging to serotype GBS III has been reported to cause both EOD and LOD, including neonatal meningitis, while ST291 belonging to serotype GBS IV is strongly associated with EOD, arthritis and septicemia^19–26^. Recent studies also reveal the increasing frequency of GBS IV and a novel GBS IV clone defined by CC17 hyper-virulent lineage in Taiwan (2010), France (2012), United States (2013), and Portugal (2014)^19,26–28^. There are few reports on the MLST of GBS in Taiwan. Tien *et al*. collected fifty isolates of invasive GBS from 2001 to 2004 and analysed them by MLST and found that the most invasive clones in adults belonging to ST1 (CC1) and serotype V, and those in infants belonged to ST17 (CC17) and serotype III^26^. This is the first study on the MLST of invasive GBS isolates in Taiwan. Our study showing a low rate of maternal colonization but high invasive diseases reflects high transmission ability and the hyper-virulence of ST17. Another report from Portugal also indicated that GBS Ia ST23 or ST24 have enhanced potential for invasive diseases, especially EOD^23^. In our study, GBS Ia ST23 and GBS Ib ST12 were the second most common pathogens, and each contributed to 7% of the invasive GBS diseases.

There has no information about the serotype distribution of maternal GBS colonisation in Taiwan before. Previous studies reported that the rate of maternal GBS colonisation was not changed and was kept around 20% in the past decade in Taiwan^7,8,13,29^. However, high maternal GBS colonization with GBS VI as the most common serotype in Taiwan is quite different from that observed in the other Western Pacific countries. Maternal GBS colonisation was around 8% in Korea with GBS III (43.8%) being the most prevalent clone, and 8.2% in Japan with GBS VIII (27–36%) being the most prevalent clone^30,31^. In China, the maternal GBS colonisation rate varied from 7.1% in Beijing, 8.2% in Dongguan, 21.8% in Hong Kong, and 32.2% in Dongying; GBS III was the predominant serotype^32–34^. In Malaysia, the predominant colonizing GBS serotype was GBS VI (30%, including non-pregnant adults)^35^. In India, the rate of GBS colonisation was 15%, where GBS III (22.2%), II (20%), and V (20%) were the most common^36^. In Europe, the maternal GBS colonisation rate varied from 6.5% to 36%, whereas GBS III was predominant, ranging from 24% to 33.2%^37^. In the U.S., the maternal colonisation rate was estimated to be 26%, with the most frequent serotypes being Ia (26.3%) and III (21.4%)^38^. Regarding genotypes, previous studies reported that GBS VI ST1 has emerged as a significant pathogen for adults in Taiwan^11,39,40^. In our study, GBS Ib ST12 (22%) was the most common colonizing clone, followed by GBS VI ST1 (17%), and GBS V ST1 (15%). There was no significant change in maternal serotype distribution between 2014 and 2017 in Taiwan. Notably, GBS VI (ST1) and GBS V (ST1) were rarely found in invasive infections of infants, suggesting that the two clones are less virulent. In our study, GBS VI ST1 (3%) and GBS V ST1 (1%) were found in invasive GBS isolates in infants. Higher genetic diversity of colonizing GBS with different serotypes and genetic characteristics from clinical isolates implies that some colonizing strains with specific characteristics may be less likely to be eradicated by IAP, more adapted to transmission, and thus more pathogenic to newborn infants.

There are two limitations in this study. First, the clinical information was collected retrospectively, and the case number of EOD was small. Second, GBS strains colonizing pregnant women were only randomly selected and examined in 2014 and 2017. However, no great variation of serotypes and genotypes were found in the two groups of isolates, as such we believe that the results remained representative of serotype distribution in the era of IAP in the pregnant women.

### Conclusions

Our study demonstrated that the distribution of serotypes of maternal GBS colonisation strains did not change between 2014 and 2017 in the era of nationwide GBS screening and IAP. GBS VI was the predominant serotype colonizing in pregnant women. However, GBS III ST17, a hyper-virulent clone, was the predominant serotype for both EOD and LOD in infants. A protein or trivalent polysaccharide conjugate vaccine targeting serotypes Ia, Ib, and III might thus protect over 90% cases of invasive GBS disease in Taiwan according to this investigation.

## Methods

### Patients and bacterial isolates

From January 2013 to June 2017, we collected 69 invasive GBS isolates from the blood of infants with invasive GBS diseases. To compare the colonizing GBS isolates, we also collected 300 GBS isolates from pregnant women at gestational age 35–37 weeks using vaginal swab in 2014 and in 2017. GBS strains were isolated in a selective BBL^™^ Lim Broth (Becton, Dickinson and Company, MD, USA) containing Columbia colistin-nalidixic acid to exclude Gram-negative bacteria, followed by GBS identification using microbial MALDI-TOF Mass Spectrometry system (Bruker Daltonics, Germany)^41^. The capsular types and genotypes of GBS isolates were identified using a multiplex polymerase chain reaction (PCR) and multilocus sequence typing (MLST), respectively. The clinical manifestations of GBS diseases were reviewed retrospectively. Respiratory failure was defined as the need for intubation and ventilator support. Leukocyte abnormality was defined as white blood cell counts less than 4,000 cells or above 20,000 cells per microliter of blood. C-reactive protein (CRP) elevation was defined as serum CRP levels more than 20 *mg*/*L*. Only the first hemogram study was recorded. This study was approved by the Institutional Review Board of Chang Gung Memorial Hospital (approval numbers: 201507336B0, 201509360B0 and 201601198A3). The informed consent was obtained from all participants, including children under 18 years of age from their parents and/or legal guardians in accordance with the guidelines of the Association for the Accreditation of Human Research Protection Programs.

### Multiplex polymerase chain reaction

A multiplex PCR assay for identifying serotypes Ia to IX of *Streptococcus agalactiae* developed by Imperi *et al*. was conducted^42^. Using a single PCR containing a mixture of 19 primer sets, the assay was used to determine GBS serotypes with the analysis of unique two- or three-band patterns on an agarose gel.

### Multilocus sequence typing

Seven housekeeping genes are used for genotyping GBS using the MLST scheme, including *adhP* (alcohol dehydrogenase gbs0054), *pheS* (phenylalanyl transfer RNA synthetase), *atr* (amino-acid transporter protein gbs0538), *glnA* (glutamine synthetase), *sdhA* (L-serine dehydratase gbs2015), *glcK* (glucose kinase gbs0518), and *tkt* (transketolase gbs0268). An online database (http://pubmlst.org/sagalactiae/)^43^ is available for assigning allele numbers and sequence types (ST). The eBURST program can be used to group isolates into clonal complexes (CCs), the members of which share at least six of seven MLST loci; otherwise, an ST can be considered a singleton^44^.

### Statistical analysis

All data were recorded and entered into a database. Analyses were performed using SPSS software, v. 22.0 (SPSS Inc., Chicago, IL, USA). A Student’s *t*-test, Chi-square test, Fisher’s exact test, or ANOVA was used when appropriate to compare proportions. All statistical analyses were two-sided, and significance was set at *P* < 0.05.

## Supplementary information

Genotype distribution based on the serotype of GBS strains in invasive diseases
