# Supplementary material for: Clinical and Microbiological Characteristics of Group B Streptococcus from Pregnant Women and Diseased Infants in Intrapartum Antibiotic Prophylaxis Era in Taiwan
Source: Sci Rep. 2019 Sep 19;9:13525. doi: 10.1038/s41598-019-49977-2 (PMC6753095; doi:10.1038/s41598-019-49977-2)
Supplement: Supplementary file 1 — Genotype distribution based on the serotype of GBS strains in invasive diseases [file 41598_2019_49977_MOESM1_ESM.docx]

**Supplementary information**

**Clinical and Microbiological Characteristics of Group B *Streptococcus* from Pregnant Women and Diseased Infants in Intrapartum Antibiotic Prophylaxis Era in Taiwan**

**Chien-Chung Lee^1^, Jen-Fu Hsu^1^, Rajendra Prasad Janapatla^2^, Chyi-Liang Chen^2^, Ying-Li Zhou^2^, Reyin Lien^1^ & Cheng-Hsun Chiu^2,3^**

^1^Division of Neonatology, Department of Pediatrics, Chang Gung Memorial Hospital, Chang Gung University College of Medicine, Taoyuan, Taiwan. ^2^Molecular Infectious Disease Research Center, Chang Gung Memorial Hospital, Taoyuan, Taiwan. ^3^Division of Pediatric Infectious Diseases, Department of Pediatrics, Chang Gung Memorial Hospital, Chang Gung University College of Medicine, Taoyuan, Taiwan.

Correspondence and requests for materials should be addressed to C.-H.Chiu (email: [chchiu@adm.cgmh.org.tw](mailto:chchiu@adm.cgmh.org.tw)).

**
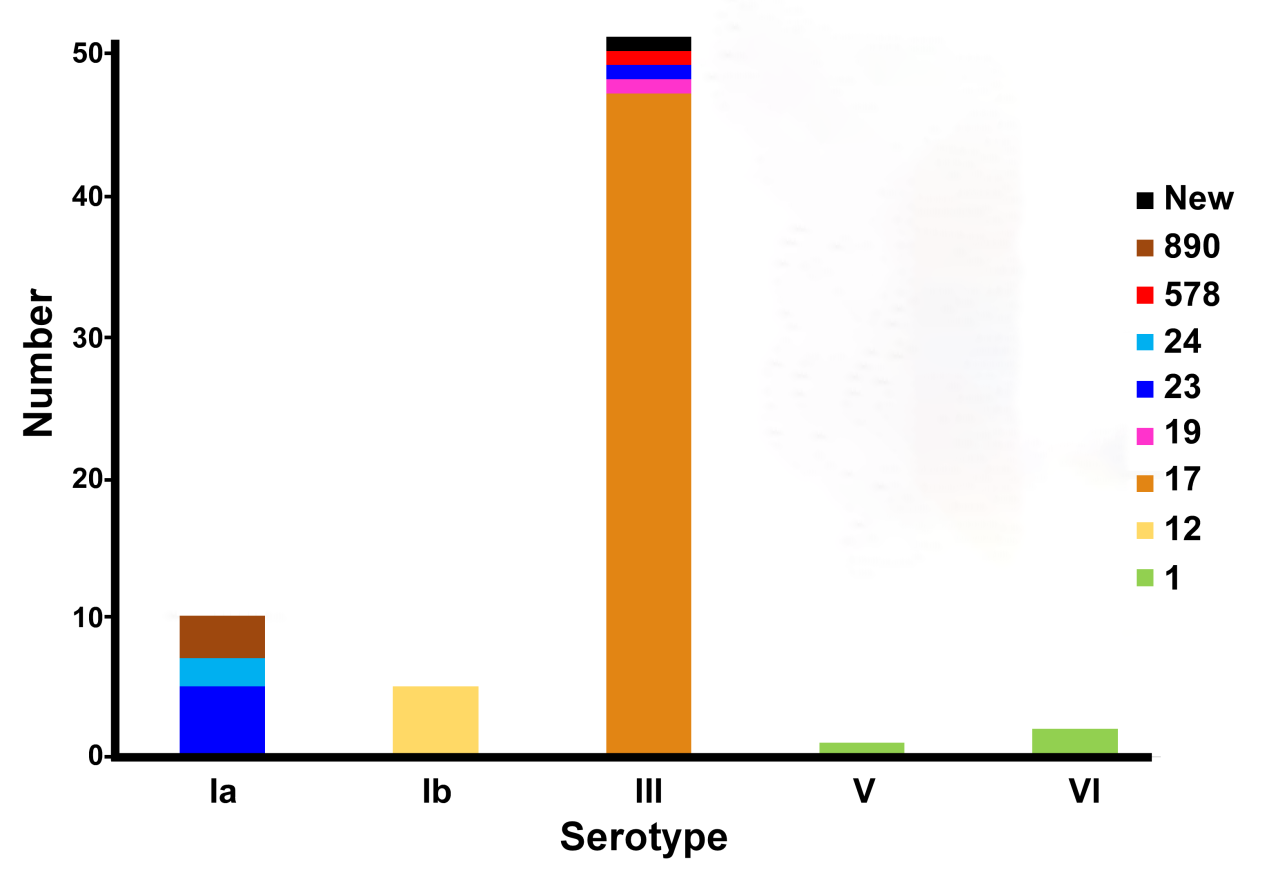
**

**Supplementary Fig. S1**

Genotype distribution based on the serotype of GBS strains in invasive diseases. The colour of the sequencing type number is shown on the right-side colour scheme.
